# Low-temperature muon spin rotation studies of the monopole charges and currents in Y doped Ho_2_Ti_2_O_7_

**DOI:** 10.1038/srep01881

**Published:** 2013-05-23

**Authors:** L. J. Chang, M. R. Lees, G. Balakrishnan, Y.-J. Kao, A. D. Hillier

**Affiliations:** 1 Department of Physics, National Cheng Kung University, Tainan 70101, Taiwan; 2 Department of Physics, University of Warwick, Coventry, CV4 7AL, United Kingdom; 3 Department of Physics and Center of Quantum Science and Engineering, National Taiwan University, Taipei 10607, Taiwan; 4 STFC Rutherford Appleton Laboratory, ISIS Facility, Didcot OX11 0QX, United Kingdom

## Abstract

In the ground state of Ho_2_Ti_2_O_7_ spin ice, the disorder of the magnetic moments follows the same rules as the proton disorder in water ice. Excitations take the form of magnetic monopoles that interact via a magnetic Coulomb interaction. Muon spin rotation has been used to probe the low-temperature magnetic behaviour in single crystal Ho_2−*x*_Y*_x_*Ti_2_O_7_ (*x* = 0, 0.1, 1, 1.6 and 2). At very low temperatures, a linear field dependence for the relaxation rate of the muon precession *λ*(*B*), that in some previous experiments on Dy_2_Ti_2_O_7_ spin ice has been associated with monopole currents, is observed in samples with *x* = 0, and 0.1. A signal from the magnetic fields penetrating into the silver sample plate due to the magnetization of the crystals is observed for all the samples containing Ho allowing us to study the unusual magnetic dynamics of Y doped spin ice.

In the spin ice materials R_2_Ti_2_O_7_ (R = Ho, Dy)^1^,^2^,^3^ a large (~ 10 *μ_B_*) magnetic moment on the R^3+^ ions giving a strong, but at low temperature almost completely screened dipole-dipole interaction, together with a local Ising-like anisotropy leads to an effective nearest-neighbour frustrated ferromagnetic interaction between the magnetic moments. The organizing principles of the magnetic ground state in spin ice, or “ice rules”, require that two R^3+^ spins should point in and two out of each elementary tetrahedron in the R_2_Ti_2_O_7_ pyrochlore lattice^2^,^4^,^5^,^6^,^7^. Excitations above the ground state manifold, which locally violate the ice rules, can be viewed as magnetic monopoles of opposite “magnetic charge” connected by Dirac strings^8^,^9^,^10^. Evidence of magnetic monopoles in spin ice has recently been observed in several experiments^11^,^12^,^13^.

Given the existence of magnetic monopoles, it is logical to consider the nature of the magnetic charges and any associated currents or “magnetricity”. Bramwell *et al.* used transverse-field muon spin-rotation (TF-*μ*SR) to investigate the magnitude and dynamics of the magnetic charge in Dy_2_Ti_2_O_7_ spin ice^14^. In these experiments the equivalence of electricity and magnetism proposed in Ref. 8 was assumed and Onsager's theory^15^, which describes the nonlinear increase with applied field in the dissociation constant of a weak electrolyte (second Wien effect), was applied to the problem of spin ice. It was argued that in spin ice, if the magnetic field *B* is changed, the relaxation of the magnetic moment *ν_μ_* occurs at the same rate as that of the monopole density and so in the weak field limit, *ν_μ_*(*B*)/*ν_μ_*(0) = *κ*(*B*)/*κ*(0) = 1 + *b*/2, where *κ* is the magnetic conductivity and 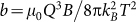 with a magnetic charge *Q*^14^. At low temperature, the fluctuating local fields lead to a de-phasing of the muon precession and an exponential decay in the oscillatory muon polarization as a function of time *t*

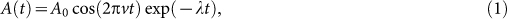
where *A*_0_ is the initial muon asymmetry, *v* = *γ_μ_B*/2*π* is the frequency of the oscillations, and *γ_μ_* is the gyromagnetic ratio. With *ν_μ_*(*B*)/*ν_μ_*(0) = *λ*(*B*)/*λ*(0) one can directly infer the magnetic monopole charge. These measurements have proven intriguing and controversial. Dunsiger *et al.*^16^ contend that the TF-*μ*SR data never takes a form where *λ* ∝ *ν* (see however^17^). It has also been suggested that the magnetic field at any muon implantation site in Dy_2_Ti_2_O_7_ is likely to take a range of values up to 0.5 T^16^,^18^,^19^. If this is the case it is difficult to understand how the fields of 1–2 mT used in Ref. 14 could lead to a precession signal. Both Dunsiger *et al.*^16^ and later Blundell^19^ have suggested that the signals seen in the *μ*SR data in Ref. 14 originate from outside the sample. In their reply to this suggestion, Bramwell *et al.*^21^ acknowledged that their experiments exploited both muons implanted in the sample (interior muons) and muons decaying outside the sample (exterior muons), with the aim of separating near and far field contributions to the signal. They went on to note that the signal at higher temperatures is dominated by muons implanted in the silver backing plate. This possibility was not discussed in their original paper^14^. Nevertheless, they continued to insist that the signal at low temperature (0.4 > *T* > 0.07 K) cannot be explained by exterior muons and that the Wien effect signal originates from muons within the sample or muons sufficiently close to the surface of the sample so as to probe the monopolar far field.

## Results

**Figure 1:**
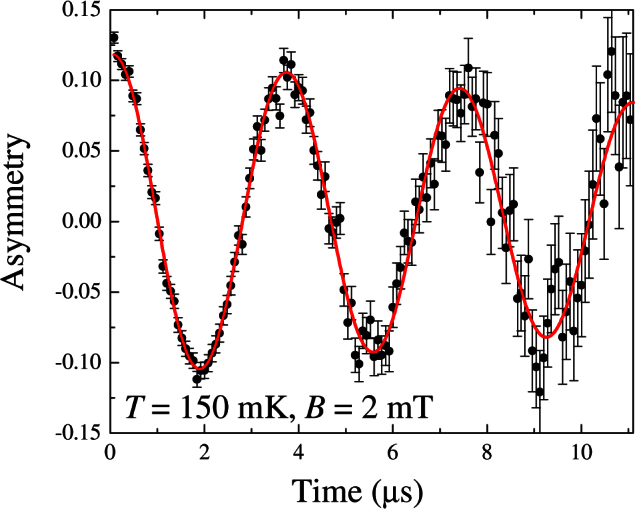
TF-*μ*SR time spectrum collected at 150 mK in a field of 2 mT for a pure Ho_2_Ti_2_O_7_ sample. These results are representative of the data collected during this study.

Fig. 1 shows a TF-*μ*SR time spectrum collected at 150 mK in a field of 2 mT for a pure Ho_2_Ti_2_O_7_ sample. This curve is representative of the data collected during this study. A rapid loss in asymmetry from an initial value of ~ 0.22 occurs outside the time window of the MuSR spectrometer^14^,^18^,^19^. The slowly relaxing component of the data were fit using Eq. 1.

**Figure 2:**
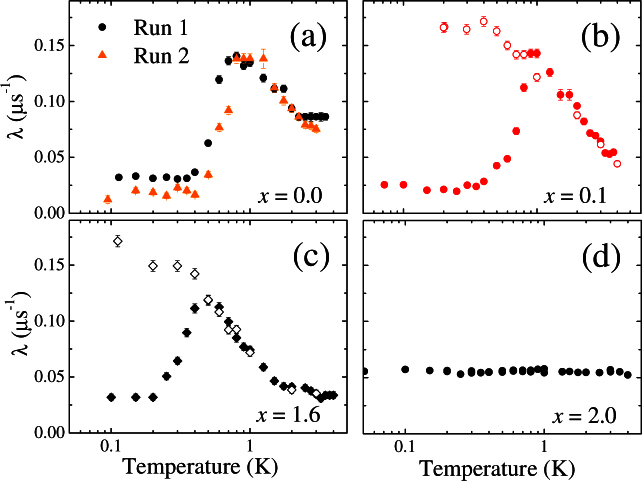
Temperature dependence of the muon relaxation rate *λ*(*T*) extracted from the fits to the TF-*μ*SR time spectra collected in 2 mT for samples of Ho_2−*x*_Y*_x_*Ti_2_O_7_ with *x* = 0, 0.1, 1.6 and 2.0. The closed symbols show the zero-field-cooled warming data and the open symbols show the field-cooled cooling data.

Fig. 2 shows the temperature dependence of the muon relaxation rate *λ*(*T*) for Ho_2−*x*_Y*_x_*Ti_2_O_7_ extracted from fits to *μ*SR time data collected in 2 mT, (see Methods and Supplementary information). For all the samples containing Ho, a nearly *T* independent *λ*(*T*) is observed at low-temperature. As the temperature is raised there is a rapid increase in *λ*(*T*) at some crossover temperature *T_CR_*. This *T_CR_* increases from ~ 0.4 K for the crystals with *x* = 1.6 and 1.0 (data not shown) to 0.5 K for the samples with *x* = 0.1 and 0.0. Above *T_CR_* the relaxation rate decreases with increasing temperature and has a similar *T* dependence for all four samples containing Ho that were studied. For two samples (*x* = 0.1 and 1.6) we also collected field-cooled-cooling data. In both cases a divergence between the zero-field-cooled warming (ZFCW) and the field-cooled cooling (FCC) curves appears at *T_CR_*. For pure Y_2_Ti_2_O_7_ a temperature independent relaxation rate is measured for the whole temperature range (0.05 to 5 K) studied.

In order to better understand the origins of these signals we have also collected relaxation data as a function of temperature in 2 mT for the pure Ho_2_Ti_2_O_7_ sample discussed above, covered with a silver foil 0.25 mm thick. This thickness of foil is expected to stop all the muons before they reach the sample. Muons implanted in silver have a negligible relaxation and so any relaxation must result from a combination of the externally applied field and/or field lines originating from the sample penetrating into the silver. The *λ*(*T*) curve obtained in this way is very similar to the signal from the pure Ho_2_Ti_2_O_7_ shown in Fig. 2a and demonstrates that at least some of the signal comes from fields within the silver, but that these fields are the result of the magnetic properties of the sample (see Supplementary information).

**Figure 3:**
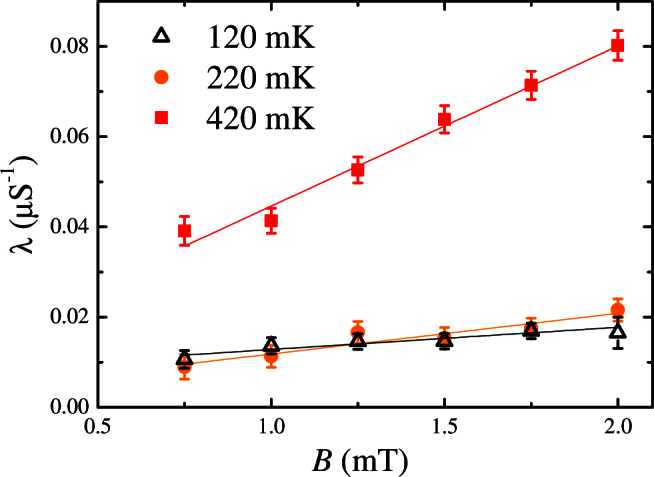
Magnetic field dependence of the muon relaxation rate *λ*(*B*) for pure Ho_2_Ti_2_O_7_ at three different temperatures. The values for *m* = (*dλ*(*B*)/*dB*)/*λ*_0_ and the effective magnetic charge *Q*_eff_ shown in Figure 4 have been obtained from the straight line fits to the data.

**Figure 4:**
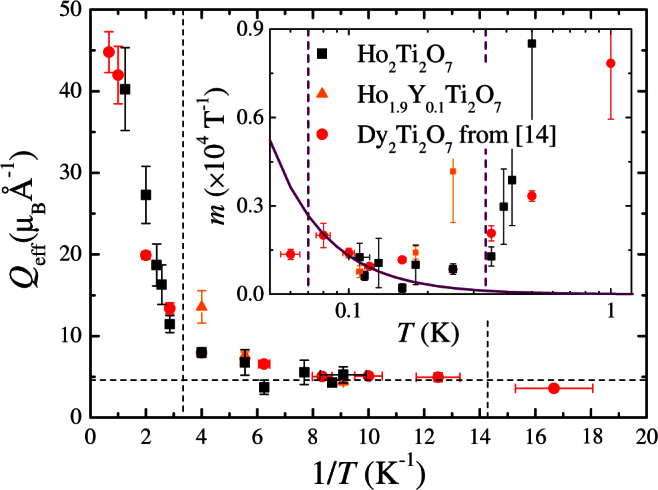
*Q*_eff_ versus 1/*T* for samples of Ho_2−*x*_Y*_x_*Ti_2_O_7_ with *x* = 0 and 0.1. The vertical dashed lines indicate the high and low temperature limits between which the Onsager theory is expected to be valid^14^ and the horizontal line marks the value for *Q*_eff_ = 4.6 *μ_B_*Å^−1^ 8. The inset shows *m*(*T*) for the same data; the solid line shows 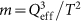 with *Q*_eff_ = 5 *μ_B_*Å^−1^. Also shown in both plots are the data of Bramwell *et al.* from Ref. 14.

As a next step we then investigated the magnetic field dependence of the muon relaxation rate. Fig. 3 shows *λ*(*B*) for a sample with *x* = 0 at selected temperatures. Studies were also made for samples with *x* = 0.1, 1, 1.6 and 2. Following Bramwell *et al.*, linear fits to the *λ*(*B*) data were made at each temperature. Using the gradient and intercept extracted from each fit, the effective magnetic charge *Q*_eff_ was obtained from *Q*_eff_ = 2.1223 *m*^1/3^*T*^2/3^, where *m* = (*dλ*(*B*)/*dB*)/*λ*_0_^14^. For samples with *x* = 0 and 0.1 the resulting values of *Q*_eff_ range from 4.5 to 7.5 *μ_B_*Å^−1^ in the temperature regime in which Onsager's theory is expected to be valid, but increase rapidly as the temperatures increase outside this range (see Fig. 4).

At high temperature, a linear field dependence for *λ*(*B*) is also observed for the two samples with a much higher yttrium doping (*x* = 1 and 1.6) but the calculated *Q*_eff_ is always greater than ~ 10 *μ_B_*Å^−1^. For *x* = 1 and 1.6 in the low-temperature regime *T* < *T_CR_* there is no systematic linear field dependence in *λ*(*B*) and no signal that can be associated with magnetricity.

We have also looked for a linear magnetic field dependence in *λ*(*B*) for the pure Ho_2_Ti_2_O_7_ sample covered in a thick (0.25 mm) silver foil. At higher temperatures *T* > *T_CR_* we observed a linear behaviour leading to a large *Q*_eff_ (i.e. *Q*_eff_ > 10 *μ_B_*Å^−1^), but at low temperatures *T* < *T_CR_* we found no signature of magnetricity and could not obtain reliable linear fits to the *λ*(*B*) data or physically acceptable values for *Q*_eff_.

## Discussion

We can draw a number of important conclusions from our work. Our results indicate that at higher temperatures, as suggested previously^16^,^19^,^21^, the dominant contribution to the *λ*(*T*) signal arises from stray fields from the magnetized spin ice that penetrate into the silver sample plate. The observation of a signal in a sample covered with thick Ag foil adds weight to this hypothesis. The sample coverage of the Ag backing plates used in our experiments was always approximately 50%. It will be interesting to explore how this signal changes as this coverage is varied. It may also be important to consider the ratio between the surface area and the volume of the spin ice in these and other experiments. Differences between the bulk and surface conductivity of water ice are well documented^20^ and it is likely that analogous processes operate in spin ice. In reply to the comments on their work, however, Bramwell *et al.*^21^ make the point that a signal from muons implanted in the sample plate may not negate the important findings of their study. Our data are consistent with the suggestion made in Ref. 21 that the Wien effect signal may arise from inside the sample or from within the Ag sample plate but at distances very close to the spin ice sample surface. We will return to this point later. First we note that the *λ*(*T*) curve for pure Ho_2_Ti_2_O_7_ follows closely the form expected for the magnetization of pure spin ice^22^ supporting the view that *λ*(*T*) reflects the magnetization in all the samples studied. This then raises an interesting question concerning the low-temperature magnetic dynamics of spin ice.

Recently there have been a number of experimental reports on the magnetic dynamics of spin ice (see for example^23^,^24^,^25^,^26^,^27^). In addition to the discussion of magnetic monopoles and the Wien effect^8^,^9^,^10^,^14^ authors have also considered the effects of thermal quenching^28^. A key component of the current theories of spin ice, is that the magnetic response at low temperatures and small applied fields is limited to monopole motion. So as the monopole density decreases the characteristic time scales become longer. This view has recently been called into question following new low-temperature AC susceptibility measurements that exhibit an activated behaviour with energy barriers that are inconsistent with the present understanding of monopoles in spin ice^25^,^29^,^30^. Our results for the *x* = 1.6 material, showing the survival of ZFCW-FCC splitting in a sample with only 15% Ho add a further twist to this puzzle. Given the large number of non-magnetic “defects” on the corners of many of the tetrahedra in this diluted material, it is not easy to attribute the slow relaxation to a low monopole density. At such low concentrations of magnetic ions even the concepts of a spin ice and monopoles are questionable.

It is conceivable that single ion physics plays a more important role in the behaviour of the diluted materials. Our diffuse neutron-scattering studies of single-crystal Ho_2−*x*_Y*_x_*Ti_2_O_7_ showed that at low temperature the scattering patterns are characteristic of a dipolar spin ice and appear to be unaffected by Y doping up to at least *x* = 1.0^31^. One possible scenario is that effects, such as distortions in the local environment due to the variation in the size of the Ho^3+^/Y^3+^ ions^32^, produce energy barriers at low-*T* that exceed the cost of an isolated monopole. The slow dynamics and the ZFCW-FCC hysteresis at low temperatures would thus cross over from a regime where this behaviour is attributed to low monopole density to a regime where it is due to exceedingly slow single ion physics. Alternatively, the long-range nature of the dipolar interactions may give rise to collective effects beyond the monopole description which introduce new energy barriers to spin flipping at very low temperatures that occur in both undiluted and diluted systems. The same qualitative form for the *λ*(*T*) data for samples with *x* = 0.1 and 1.6 indicate that additional ingredients may be required to explain the low *T* behaviour in spin ice and that further studies on diluted samples are needed to fully understand the role played by factors such as impurities, dislocations, and surface effects on the low-temperature dynamics of spin ice.

Returning to the question of magnetricity in spin ice we note that in our *μ*SR data the low-temperature signal that has previously been interpreted as a signature of magnetricity is seen in the *x* = 0 and 0.1 samples and is not observed in the more dilute Ho_2−*x*_Y*_x_*Ti_2_O_7_ materials. Within the *T* range indicated by the dashed lines in Fig. 4, where the theory presented by Bramwell *et al.* is expected to be valid, the value of *Q*_eff_ agrees with expectations. Following Blundell^19^ we also plot *m* versus *T*. We see that the expected *m* ∝ *T*^−2^ only holds for the same narrow *T* range. Our experiments, including two separate runs on pure Ho_2_Ti_2_O_7_ carried out three months apart, demonstrate the reproducibility of the data (see Fig. 2a). A realignment of the Ho_2_Ti_2_O_7_ disks between runs also shows that the results are not particularly sensitive to the exact details of the sample geometry. Our results for the samples with a higher Y content and with the thick Ag foil demonstrate that the behaviour cannot be attributed to instrumental effects. The samples were made at Warwick^33^ and are Ho rather than Dy based pyrochlores, eliminating the possibility of material specific results.

In summary transverse-field *μ*SR experiments on Ho_2−*x*_Y*_x_*Ti_2_O_7_, including measurements on non-magnetic Y_2_Ti_2_O_7_ and a sample of Ho_2_Ti_2_O_7_ covered in thick silver foil, suggest that the majority signal in the *λ*(*T*) response comes from stray fields due the sample magnetization penetrating into the silver sample plate^16^,^19^. The results for Ho_2_Ti_2_O_7_ are comparable with those observed for Dy_2_Ti_2_O_7_. The low-temperature (*T* < *T_CR_*) linear field dependence in *λ*(*B*) is only observed in samples with *x* = 0 and 0.1. In this low-temperature regime the value of *Q*_eff_ agrees quantitatively with the theory presented in Ref. 14. The low-temperature hysteresis in *λ*(*T*) for the magnetically dilute material (*x* = 1.6) appears inconsistent with the current understanding of monopoles in spin ice.

## Methods

Single crystals of Ho_2−*x*_Y*_x_*Ti_2_O_7_ (*x* = 0, 0.1, 1, 1.6 and 2) were grown in an image furnace using the floating zone technique^33^. The single crystal disks were glued on to a silver plate and covered with a thin (0.01 mm) sheet of silver foil to improve thermal conductivity. The plate was then attached to the cold stage of an Oxford Instruments ^3^He/^4^He dilution refrigerator. Transverse-field muon spin-rotation experiments were performed using the MuSR spectrometer at the ISIS pulsed muon facility, Rutherford Appleton Laboratory, UK. The magnetic field was applied along the [001] direction, perpendicular to the initial direction of the muon spin polarization which was along the [110] axis. Measurements were carried out as a function of applied field at fixed temperature and as a function of temperature in a fixed magnetic field. See Supplementary information for full details of the measurement protocols.

## Author Contributions

L.J.C. and M.R.L. conceived of the project. G.B. prepared the samples. A.D.H., L.J.C. and M.R.L. planned and carried out the experiments. M.R.L., A.D.H., Y.J.K. and L.J.C. helped to analyse the data, draft the paper, and prepare the figures. All the authors reviewed the manuscript.

## Supplementary Material

Low-temperature muon spin rotation studies of the monopole charges and currents in Y doped Ho_2_Ti_2_O_7_ Supplementary Information

## References

[b1] Ramirez A. P., Hayashi A., Cava R. J., Siddharthan R. & Shastry B. S. Zero-point entropy in ‘spin ice’. Nature 399, 333–335 (1999).

[b2] Harris M. J., Bramwell S. T., McMorrow D. F., Zeiske T. & Godfrey K. W. Geometrical frustration in the ferromagnetic pyrochlore Ho_2_Ti_2_O_7_. Phys. Rev. Lett. 79, 2554–2557 (1997).

[b3] Bramwell S. T. & Gingras M. J. P. Spin ice state in frustrated magnetic pyrochlore materials. Science 294, 1495–1501 (2001). 11711667 10.1126/science.1064761

[b4] Bramwell S. T., & Harris M. J. Frustration in Ising-type spin models on the pyrochlore lattice. J. Phys.: Condens. Matter 10, L215–L220 (1998).

[b5] den Hertog B. C. & Gingras M. J. P. Dipolar interactions and origin of spin ice in Ising pyrochlore magnets. Phys. Rev. Lett. 84, 3430–3433 (2000). 11019107 10.1103/PhysRevLett.84.3430

[b6] Yavors'kii T., Fennell T., Gingras M. J. P. & Bramwell S. T. Dy_2_Ti_2_O_7_ spin ice: a test case for emergent clusters in a frustrated magnet. Phys. Rev. Lett. 101, 037204 (2008). 18764286 10.1103/PhysRevLett.101.037204

[b7] Isakov S. V., Moessner R. & Sondhi S. L. Why spin ice obeys the ice rules. Phys. Rev. Lett. 95, 217201 (2005). 16384174 10.1103/PhysRevLett.95.217201

[b8] Castelnovo C., Moessner R. & Sondhi S. L. Magnetic monopoles in spin ice. Nature 451, 42–45 (2008). 18172493 10.1038/nature06433

[b9] Ryzhkin I. A. Magnetic relaxation in rare-earth oxide pyrochlores. J. Exp. Theor. Phys. 101, 481–486 (2005).

[b10] Jaubert L. D. C. & Holdsworth P. C. W. Signature of magnetic monopole and Dirac string dynamics in spin ice. Nature Phys. 5, 258–261 (2009).

[b11] Fennell T. *et al.* Magnetic Coulomb phase in the spin ice Ho_2_Ti_2_O_7_. Science 326, 415–417 (2009). 19729619 10.1126/science.1177582

[b12] Morris D. J. P. *et al.* Dirac strings and magnetic monopoles in the spin Ice Dy_2_Ti_2_O_7_. Science 326, 411–414 (2009). 19729617 10.1126/science.1178868

[b13] Kadowaki H. *et al.* Observation of magnetic monopoles in spin ice. J. Phys. Soc. Jpn. 78, 103706 (2009).

[b14] Bramwell S. T. *et al.* Measurement of the charge and current of magnetic monopoles in spin ice. Nature 461, 956–959 (2009). 19829376 10.1038/nature08500

[b15] Onsager L. Deviations from Ohm′s law in weak electrolytes. J. Chem. Phys. 2, 599–615 (1934).

[b16] Dunsiger S. R. *et al.* Spin ice: magnetic excitations without monopole signatures using muon spin rotation. Phys. Rev. Lett. 107, 207207 (2011). 22181768 10.1103/PhysRevLett.107.207207

[b17] Bramwell S. T. Generalized longitudinal susceptibility for magnetic monopoles in spin ice. Phil. Trans. R. Soc. A. 370, 5738–5766 (2012). 23166378 10.1098/rsta.2011.0596 PMC3497062

[b18] Lago J., Blundell S. J. & Baines C. *μ*SR investigation of spin dynamics in the spin-ice material Dy_2_Ti_2_O_7_. J. Phys.: Condens. Matter 19, 326210 (2007).

[b19] Blundell S. J. Monopoles, magnetricity, and the stray field from spin ice. Phys. Rev. Lett. 108, 147601 (2012). 22540822 10.1103/PhysRevLett.108.147601

[b20] Petrenko V. F. & Whitworth R. W. Physics of Ice (Oxford University Press, Oxford, 1999).

[b21] Bramwell S. T. & Giblin S. R. Comment on ‘Spin ice: magnetic excitations without monopole signature using *μ*SR’ (arXiv:1110.0877). arXiv:1111.4168.

[b22] Snyder J. *et al.* Low-temperature spin freezing in the Dy_2_Ti_2_O_7_ spin ice. Phys. Rev. B 69, 064414 (2004).

[b23] Giblin S. R., Bramwell S. T., Holdsworth P. C. W., Prabhakaran D. & Terry I. Creation and measurement of long-lived magnetic monopole currents in spin ice. Nature Phys. 7, 252–258 (2011).

[b24] Slobinsky D. *et al.* Unconventional magnetization processes and thermal runaway in spin-ice Dy_2_Ti_2_O_7_. Phys. Rev. Lett. 105, 267205 (2010). 21231712 10.1103/PhysRevLett.105.267205

[b25] Yaraskavitch L. R. *et al.* Spin dynamics in the frozen state of the dipolar spin ice material Dy_2_Ti_2_O_7_. Phys. Rev. B 85, 020410 (2012).

[b26] Erfanifam S. *et al.* Intrinsic and extrinsic nonstationary field-driven processes in the spin-ice compound Dy_2_Ti_2_O_7_. Phys. Rev. B 84, 220404 (2011).

[b27] Petrenko O. A., Lees M. R. & Balakrishnan G. Titanium pyrochlore magnets: how much can be learned from magnetization measurements? J. Phys.: Condens. Matter 23, 164218 (2011). 21471629 10.1088/0953-8984/23/16/164218

[b28] Castelnovo C., Moessner R. & Sondhi S. L. Thermal quenches in spin ice. Phys. Rev. Lett. 104, 107201 (2010). 20366451 10.1103/PhysRevLett.104.107201

[b29] Matsuhira K. *et al.* Spin dynamics at very low temperature in spin ice Dy_2_Ti_2_O_7_. J. Phys. Soc. Jpn. 80, 123711 (2011).

[b30] Quilliam J. A., Yaraskavitch L. R., Dabkowska H. A., Gaulin B. D. & Kycia J. B. Dynamics of the magnetic susceptibility deep in the Coulomb phase of the dipolar spin ice material Ho_2_Ti_2_O_7_. Phys. Rev. B 83, 094424 (2011).

[b31] Chang L. J. *et al.* Magnetic correlations in the spin ice Ho_2*x*_Y*_x_*Ti_2_O_7_ as revealed by neutron polarization analysis. Phys. Rev. B 82, 172403 (2010).

[b32] Snyder J. *et al.* Quantum and thermal spin relaxation in the diluted spin ice Dy_2*x*_M*_x_*Ti_2_O_7_ (M = Lu, Y). Phys. Rev. B 70, 184431 (2004).

[b33] Balakrishnan G., Petrenko O. A., Lees M. R. & Paul D. M. Single crystal growth of rare earth titanate pyrochlores. J. Phys.: Condens. Matter 10, L723–L725 (1998).

