# Supplementary material for: Low-temperature muon spin rotation studies of the monopole charges and currents in Y doped Ho2Ti2O7
Source: Sci Rep. 2013 May 23;3:1881. doi: 10.1038/srep01881 (PMC3662007; doi:10.1038/srep01881)
Supplement: Low-temperature muon spin rotation studies of the monopole charges and currents in Y doped Ho2Ti2O7 — Supplementary Information [file srep01881-s1.pdf]

Low-temperature muon spin rotation studies of the monopole charges and currents in Y doped $Ho_2Ti_2O_7$

L. J. Chang[1], M. R. Lees[2], G. Balakrishnan[2],
Y. -J. Kao[3], and A. D. Hillier[4]

[1]Department of Physics, National Cheng Kung University,
Tainan 70101, Taiwan
[2]Department of Physics, University of Warwick,
Coventry, CV4 7AL, United Kingdom
[3]Department of Physics and Center of Quantum Science and
Engineering, National Taiwan University, Taipei 10607, Taiwan
[4]STFC Rutherford Appleton Laboratory, ISIS Facility,
Didcot OX11 0QX, United Kingdom

Experimental Methods

Sample Preparation

Single crystals of $Ho_{2-x}Y_xTi_2O_7$ ($x = 0$, 0.1, 1, 1.6 and 2) were grown in an image furnace using the floating zone technique [1]. The cylindrical crystals were cut into circular disks ~ 6 mm in diameter and ~ 1 mm thick. These disks were oriented using the Laue x-ray diffraction technique and then glued, using GE varnish, in a circular pattern on to a silver sample plate as shown in Fig. 1. The samples were covered with a thin (0.01 mm) sheet of silver foil to improve thermal conductivity and mounted on the cold stage of an Oxford Instruments $^3He/^4He$ dilution refrigerator.

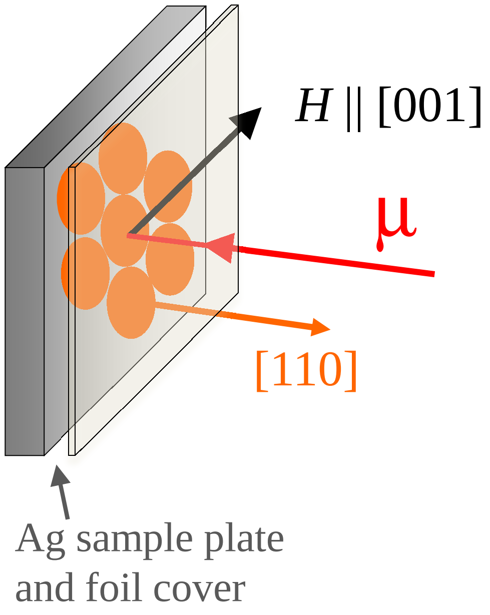

Figure 1: Schematic of the experimental sample geometry used for the transverse-field μSR measurements. The single crystal disks were glued to a silver sample plate with GE varnish. The transverse field (TF) H was applied along a [001] direction perpendicular to the initial direction of the muon spin polarization which was along a [110] axis.

μSR Experiments

Muon spin rotation (μSR) experiments were performed using the MuSR spectrometer at the ISIS pulsed muon facility, Rutherford Appleton Laboratory, United Kingdom.

For the magnetic field sweeps at fixed temperature the samples were zero-field cooled to a temperature well below the eventual measuring temperature, thermalised, and then slowly warmed in zero field to the required measuring temperature. A transverse external field was then applied. For these measurements each field point took approximately 15 minutes to collect. At the end of each field sweep the magnetic field was reduced to zero and the sample warmed to 4 K. Note, during zero-field cooling, the stray fields at the sample position were cancelled to less than 3 μT by three pairs of coils forming an active compensation system.

The temperature sweeps were always made following a field sweep measurement at base temperature. This means that in practice the samples were zero-field-cooled to the base temperature of the cryostat, thermalised and a field of 2 mT was applied in steps of 0.25 mT over a period of at least two hours. Data were then collected in zero-field-cooled warming (ZFCW) mode by warming the sample to each measuring temperature up to maximum of 4 K and then in field-cooled cooling (FCC) mode on subsequent cooling to each measuring temperature. Each point in these temperature scans took around 15 minutes to collect. Due to the large low-temperature hyperfine contribution to the specific heat for the samples containing holmium, the effective base temperature of the dilution refrigerator for these samples was limited to 100 mK.

$Ho_2Ti_2O_7$ covered in thick silver foil

Muon spin rotation spectra for a sample of pure $Ho_2Ti_2O_7$ covered with a silver foil 0.25 mm thick were collected at fixed temperature in 2 mT. The temperature dependence of the muon relaxation rate $\lambda(T)$ extracted from fits to this data are shown in Fig. 2.

For the low-temperature data ($T < T_{CR}$) the data were fit using

$$A(t) = A_0 \cos(2\pi \upsilon t) \exp(-\lambda t), \tag{1}$$

where A_0 is the initial muon asymmetry, $\upsilon = \gamma_\mu B/2\pi$ is the frequency of the oscillations, and γ_μ is the gyromagnetic ratio.

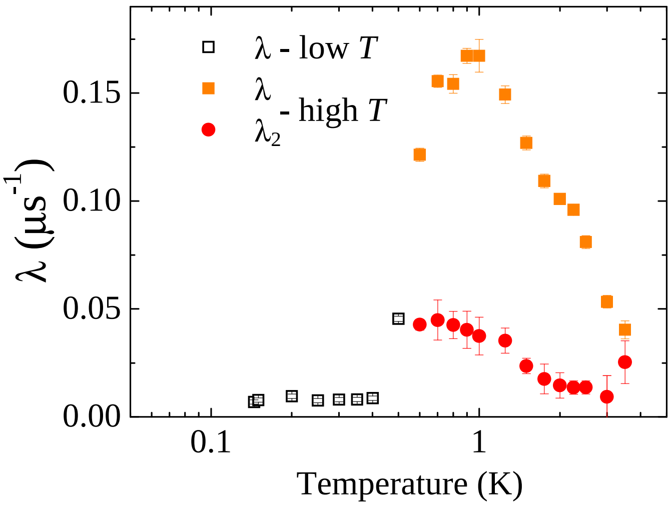

Figure 2: Temperature dependence of the muon relaxation rate $\lambda(T)$ extracted from fits to the TF-μSR time spectra collected in 2 mT during ZFCW for a sample of $Ho_2Ti_2O_7$ covered with 0.25 mm thick silver foil. At low temperature a fit using Equation 1 gives λ. At higher temperature a two component fit using Equation 2 gives λ and λ_2.

In order to obtain satisfactory fits to the data above T_{CR} the modified expression.

$$A(t) = A_0 \cos(2\pi \upsilon t) \exp(-\lambda t) + A_2 \exp(-\lambda_2 t) \tag{2}$$

was used. The additional $A_2 \exp(-\lambda_2 t)$ term is required to take account of the larger range stray fields within the thick silver foil. This is because the muon facility at ISIS has a significant momentum bite and so the implantation distance for the lower energy muons will be less than those with a higher energy.

References

[1] Balakrishnan, G., Petrenko, O. A., Lees, M. R. & Paul, D. M. Single crystal growth of rare earth titanate pyrochlores. *J. Phys.: Condens. Matter* **10**, L723-L725 (1998).
